# Fifty‐eight‐year‐old female with abdominal migraine: A rare cause of episodic gastrointestinal disturbance in adults

**DOI:** 10.1002/ccr3.2891

**Published:** 2020-05-06

**Authors:** Rehan Karmali, Gordon Hall‐Wurst

**Affiliations:** ^1^ Orange Regional Medical Center ‐ Internal Medicine Middletown New York; ^2^ Touro College of Osteopathic Medicine Middletown New York

**Keywords:** abdominal migraine, adult, childhood periodic syndrome, cyclic vomiting syndrome

## Abstract

Abdominal migraine (AM) is a predominantly pediatric condition characterized by erratic episodes of abdominal pain, nausea, and vomiting with spontaneous periodic relief. It should be considered as a differential diagnosis in symptomatic adults.

## Graphical abstract

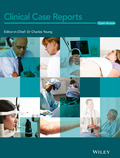

## 1 INTRODUCTION

Abdominal migraine (AM) is a predominantly pediatric condition first described in 1922 as “periodic function epigastralgia.”^1^ It is characterized by erratic bouts of acute abdominal pain that are typically associated with headache and vomiting and may also include anorexia, photophobia, flushing, and pallor.^2^ This condition is often worked up and treated for irritable bowel syndrome, functional dyspepsia, and food allergies. Children with AMs usually grow out of the condition but some may grow up to experience migraine headaches and recurrent abdominal pain as adults.^3^ The diagnostic criteria for AM have been established in children,^4^, ^5^ and our review of the literature shows evidence of AM being diagnosed in adults.^6^, ^7^, ^8^, ^9^, ^10^, ^11^, ^12^, ^13^, ^14^, ^15^, ^16^, ^17^ Here, we present the case of a 58‐year‐old female who met the established diagnostic criteria for AM following three hospital readmissions and extensive workup to rule out other etiologies.

## 2 CASE PRESENTATION

The patient is a 58‐year‐old female who presented with multiple episodes of coffee ground emesis in August 2019. These episodes were preceded by nearly 20 episodes of greenish nonbloody emesis that started 3 days prior to admission. She has a 7‐month history of 2‐ to 3‐day episodes dating back to February 2019 which are accompanied by abdominal pain, nausea, and loss of appetite requiring three admissions to the hospital. She described her abdominal pain as moderate intensity, continuous, dull, and predominantly midline without radiation. Her pain was associated with a mild headache but no visual changes or preceding aura. On physical examination, her abdomen was soft, mildly distended with epigastric and periumbilical tenderness and no guarding or rebound. She had periodic relief from her symptoms but given their unpredictability she was dependent on her 80‐year‐old mother for assistance with her activities of daily living. She denied any triggers for her symptoms or any fever, chills, constipation, diarrhea, weight loss, or skin changes. Her family history was noncontributory, and she did not drink alcohol, smoke, or use recreational drugs. In her previous visits, she typically improved while in the hospital, but worsened when she returned home. Thus far, she had failed treatment with omeprazole, ondansetron, sucralfate, meclizine, scopolamine, and topiramate. Her past medical history is significant for coronary artery disease and placement of a drug‐eluting stent in July 2019 for which she was taking dual antiplatelet therapy with aspirin and ticagrelor. In the emergency room, she was hypokalemic and hyperglycemic with an elevated anion gap, ketonuria, and mixed metabolic acid‐base disorder on venous blood gas (Table 1). She received potassium chloride, insulin lantus and sliding scale, D5 and half normal saline for suspected diabetic ketoacidosis and her anion gap, beta‐hydroxybutyrate, potassium, and glucose all improved to within normal limits. Moreover, given her low hemoglobin and coffee ground emesis, she was suspected to have an upper gastrointestinal (UGI) bleed as a possible side effect of her cardiac medications and admitted for starvation ketoacidosis. Aspirin was discontinued upon admission as a precaution and she was started on intravenous fluids and antiemetics. She was not taking any NSAIDs.

**TABLE 1:** Laboratories on presentation

**CBC with differential**		**General chemistry and coagulation profile**	
WBC	9.8 × 10^3^/µL	Sodium	140 mEq/L
RBC	4.18 × 10^6^/µL	Potassium	3.0 mEq/L
Hemoglobin	11.1 g/dL	Chloride	102 mEq/L
Hematocrit	33.5%	CO2	18 mEq/L
MCV	80.1 fL	BUN	25 mg/dL
MCH	26.6 pg	Glucose	264 mg/dL
MCHC	33.1 g/dL	Calcium	9.9 mg/dL
RDW	15.3%	Creatinine, serum	1.26 mg/dL
Platelets	302 × 10^3^/µL	Total bilirubin	1.5 mg/dL
Abs neutrophil count	8261 cells/mm^3^	Bilirubin, Direct	0.13 mg/dL
Neutrophils relative	84.3%	Bilirubin indirect	1.37 mg/dL
Lymphocytes relative	8.6%	Albumin	4.5 g/dL
Monocytes relative	6.7%	Total protein	7.6 g/dL
Eosinophils relative	0.0%	Alkaline Phosphatase	96 U/L
Basophils relative	0.1%	AST	15 U/L
IG relative	0.3%	ALT	14 U/L
Neutrophils absolute	8.2 × 10^3^/µL	Anion Gap	20 mEq/L
Lymphocytes absolute	0.8 × 10^3^/µL	eGFR non‐African American	43.6 mL/min/1.732 m^2^
Monocytes absolute	0.7 × 10^3^/µL	Protime	11.9 s
Eosinophils absolute	0.0 × 10^3^/µL	INR	1.03
Basophils absolute	0.0 × 10^3^/µL	aPTT	29.4 s
**Cardiac biomarkers**		**Venous blood gas**	
Total CK	69 U/L	pH venous	7.412
Troponin I	0.03 ng/mL	pCO2 venous	29.8 mm Hg
		pO2 venous	46.4 mm Hg
		HCO3 venous	18.6 mmol/L
**Special chemistry**		Base excess venous	−4.9 mmol/L
Beta hydroxybutyrate	2.25 mmol/L	CO2 total venous	19.5 mmol/L
		O2 sat venous	81%

## 3 DIFFERENTIAL DIAGNOSIS AND INVESTIGATIONS

### 3.1 Gastroenterology

Gastroenterology (GI) was consulted for her case. Differential diagnoses included esophagitis, gastritis, peptic ulcer disease, celiac disease, small bowel obstruction, inflammatory bowel disease, pancreatitis, irritable bowel syndrome (IBS), lactose intolerance, chronic hepatitis, hernia, appendicitis or intussusception, intestinal polyps, or gallbladder disease. A computed tomography (CT) scan of her abdomen and pelvis showed a small hiatal hernia with distal esophageal thickening, small uncomplicated 1.8 cm splenic artery aneurysm and no acute changes in the bowel and mesentery. The finding of distal esophageal thickening was followed up via an esophagogastroduodenoscopy (EGD). It did not show any ulcers, strictures, or signs of UGI bleed, and subsequent biopsies were negative for any malignancies. IBS was ruled out since the patient moved her bowels regularly without any constipation or diarrhea. Given these findings, the GI etiology for her symptoms was deemed unlikely at this time and she was managed with intravenous omeprazole, ondansetron, and PO sucralfate with limited success.

### 3.2 Endocrine

The patient had a history of noninsulin‐dependent diabetes mellitus well controlled with metformin and glipizide with a Hemoglobin A1C of 6.2. Diabetic gastroparesis was suspected, but follow‐up nuclear motility scan and gastric emptying studies were normal. Given their side effect profile, metformin and glipizide were discontinued and replaced by sliding scale insulin but this did not improve the patient's symptoms. Hence, an endocrine etiology for her symptoms was deemed unlikely.

### 3.3 Neurology

Neurology was also consulted for her case as the patient had a history of a cerebellar stroke in May 2019 with residual mild left lower extremity ataxia. Her presenting symptoms were unusual in the setting of a possible new cerebellar stroke; however, hydrocephalus, posterior fossa disorders, intracranial hypertension, and new cerebrovascular injury were suspected. Follow‐up MRI of the brain showed only showed a stable chronic cerebellar infarct with no evidence of hydrocephalus, mass effect, acute infarct, or hemorrhage. Hence, a neurologic etiology for her symptoms was also ruled out.

### 3.4 Infection

Parasitic infections, *Helicobacter pylori (H. pylori)* gastritis and pneumonia, were among infectious etiologies that could explain the patient's symptoms. However, blood cultures and urinalysis were normal. CBC did not show an elevated white blood cell count. As mentioned previously, EGD with biopsy did not show any signs of *H. pylori* gastritis. Lastly, a chest X‐ray showed no acute pulmonary process or any signs of pneumonia.

### 3.5 Cardiovascular

Given recent stent placement, she was worked up for a myocardial infarction but her EKG was normal and troponins were negative. Furthermore, the continuous nature of her pain suggested revisiting a possible GI etiology.

## 4 ABDOMINAL MIGRAINE DIAGNOSIS AND TREATMENT

Thus, a multidisciplinary discussion of this patient was held since all the aforementioned etiologies were deemed unlikely. During the hospital course, the patient had periodic relief from her symptoms similar to what she experienced at home. Her unpredictable paroxysms of debilitating nausea, vomiting, and abdominal pain in the absence of another identifiable medical disorder led to the diagnosis of AM being reached. The criteria outlined by the International Classification of Headache Disorders, 2nd Edition (ICHD‐II),^4^ and the Rome III criteria^5^ for AM were utilized to reach this diagnosis. The patient had previously failed therapy with omeprazole, ondansetron, sucralfate, meclizine, scopolamine, and topiramate. Given the absence of clear migraine headaches in this patient or in her family history, the decision was made treat her emesis‐dominant AM symptoms with a trial of prochlorperazine as opposed to a triptan. While not first line, prochlorperazine has been shown to have antiemetic properties including in an AM patient.^8^, ^18^, ^19^ This gave her sustained relief from her symptoms, and she was able to tolerate a regular diet and discharged. Per recent outpatient follow‐up she remains symptom free.

## 5 DISCUSSION

The adult variant of AM appears to share many symptoms with the pediatric as is evidenced in this case as well as several others reported in the literature.^6^, ^7^, ^8^, ^9^, ^10^, ^11^, ^12^, ^13^, ^14^, ^15^, ^16^ Patients with AMs typically present with onset of nausea, abdominal pain, vomiting, and photophobia. There are no relevant laboratory markers associated with this condition so obtaining a solid medical history is crucial to the diagnosis. Tables 2 and 3 summarize the established pediatric diagnostic criteria for AMs. It should be noticed that symptoms tend to disappear spontaneously and only seem to appear during an acute AM attack. As such, patients are typically free of symptoms in the time periods between attacks. Attacks usually last between 2‐72 hours if not successfully treated. Typically, patients have at least two associated symptoms of anorexia, nausea, vomiting, and pallor during attacks. Some patients experience dull pain in the umbilical area that can become severe. Patients with severe symptoms are at risk for hypovolemic shock and acid‐base abnormalities due to repeated emesis. Associated hypokalemia can cause dangerously irregular heart rhythms and must be managed immediately. Additional complications include esophageal cancer, GERD, and ulcers in addition to starvation ketosis and elevated anion gap metabolic acidosis as seen in our patient.

**TABLE 2:** ICHD‐2 criteria^4^

A. At least five attacks fulfilling criteria B‐D B. Attacks of abdominal pain lasting 1‐72 h (untreated or unsuccessfully treated) C. Abdominal pain has all of the following characteristics: Midline location Periumbilical or poorly localized Dull or “just sore” quality Moderate or severe intensity D. During abdominal pain, at least two of the following: Anorexia Nausea Vomiting Pallor E. Not attributable to another disorder

**TABLE 3:** American College of Gastroenterology Rome III criteria ^5^

Paroxysmal episodes of intense, acute periumbilical pain with intervening periods of usual health lasting weeks to months The pain interferes with normal activities The pain is associated with two or more of the following: Anorexia Nausea Vomiting Headache Photophobia Pallor No evidence of an inflammatory, anatomic, metabolic, or neoplastic process considered that explains the subject's symptoms. The criteria must have been fulfilled two or more times in the preceding 12 mo

Abdominal migraine is poorly understood and few different hypotheses exist to explain its pathophysiology. A visceral hyperalgia hypothesis suggests early indistinct environmental stressors to the enteric nervous system can cause altered release of gut hormones such as serotonin impairing intestinal secretions and motility.^3^ AM patients also tend to have slower gastric emptying rates, increased gut permeability, abnormal gut motility, and enteric electrolyte imbalances which together may underlie symptomatology.^20^, ^21^ In addition, the strong connection between the enteric nervous system and CNS could suggest a pathway for migraine headaches to follow AMs. However, migraine headaches and abdominal symptoms are not always linked in adults^15^ as witnessed in our patient who only had a mild headache without classic migraine headache symptoms or a contributing family history. Furthermore, Tables 2 and 3 show that established pediatric AM criteria do not require migraine headaches to be present in order to confirm the diagnosis. To some extent, this may explain why our patient was resistant to many of the medications typically used to treat AM with associated migraine headaches. An alternate theory about AM is that it is a neuroendocrine driven “brain‐gut disorder” wherein central changes in neurotransmitter release may alter vagal control of the GI tract.^22^ There is not one clear cause of AMs and it is conceivable that more than one of these hypotheses could be involved in the pathophysiology along with other external and internal factors like genetics, diet, lifestyle, stress, and environmental exposures.^3^, ^23^ This is in accord with AM being a clinical diagnosis of exclusion.

Evidence in the literature about the treatment of AM in adults is limited. A diet low in amines and high in fiber may help lower the frequency of the migraines and abdominal pain.^23^ Calcium channel blockers,^12^, ^16^, ^24^ beta‐blockers^14^ topiramate,^10^, ^24^ valproate,^11^, ^25^ metoclopramide and NSAIDS,^7^ antihistamines^12^, ^26^ and triptans^8^, ^10^, ^11^, ^24^, ^26^, ^27^, ^28^ have shown efficacy as both prophylactic and abortive agents. Prochlorperazine previously demonstrated antiemetic properties including in a case of AM.^8^, ^18^, ^19^ The chemoreceptor trigger zone in the brain has dopamine (D2) receptors that are antagonized by prochlorperazine.^29^ Hence, its efficacy in this patient supports the neuroendocrine hypothesis for AMs in that its central dopamine antagonism may have altered the downstream neuroendocrine effects on the gut, thereby ameliorating the patient's symptoms. This may have implications in AM patients who do not have accompanying migraine headaches and have symptoms dominated by nausea and emesis. However, further investigations in adult AM patients are required to support this theory.

Irregular patterns of nausea and vomiting are also known to be a product of psychiatric illnesses such as depression, anxiety, conversion disorder, and obsessive‐compulsive disorder.^30^ Our index of suspicion was not high for a psychiatric etiology for this patient as she did not have a history of any of these conditions and did not display associated symptoms across her admissions. Nonetheless given the absence of an organic pathology and successful treatment with the antipsychotic prochlorperazine, a full psychiatric evaluation may have been of benefit to uncover possible underlying psychiatric disease.

Cyclic vomiting syndrome (CVS) is a similar condition to AM as it too is a diagnosis of exclusion characterized by recurrent episodes of vomiting with intervals of baseline health.^31^ It requires three or more stereotypical episodes lasting less than a week in the prior year and two within the past 6 months that occur at least 1 week apart.^32^ Symptoms of abdominal pain, vomiting, and headaches overlap between the two conditions; hence, our patient may have broadly met the diagnostic criteria for CVS. However, while CVS is emesis‐predominant, up to 81% of patients also experience abdominal pain and while AM is pain‐predominant, up to 72% of patients also experience vomiting.^33^ Unlike AM, CVS episodes have an almost circadian pattern of symptom onset in the early morning hours prior to sunrise with mean episode duration of 6 days.^22^, ^31^, ^34^ Our patient's episodes were 2‐3 days in length which is shorter than the mean duration of CVS episodes and diagnostically closer to AM episode length.^4^ Furthermore, she typically presented in the late mornings or early afternoons. Her moderate intensity, midepigastric and dull abdominal pain pattern align closer with AM and she was treated with a medication, prochlorperazine, that has prevented emesis in diagnosed AM cases.^8^, ^18^, ^19^

## 6 CONCLUSION

Abdominal migraine is a rare, multifactorial disease characterized by unpredictable paroxysms of abdominal pain, nausea, and vomiting. It is primarily a pediatric condition but has been known to be diagnosed in adults. Our review of this case suggests that the adult variant of the condition shares key features with pediatric cases and should be considered as a differential diagnosis in adults with similar symptomatology. There is limited evidence about various abortive and prophylactic agent to treat underlying AM in adults. Prochlorperazine may be considered as a potential therapeutic for treatment‐resistant AM patients.

## CONFLICT OF INTEREST

None declared.

## AUTHOR CONTRIBUTION

RK: initiated this case report, performed literature review, and wrote first and final manuscript draft. GH: performed literature review and contributed to contents of this manuscript.

## CONSENT STATEMENT

Patient provided written consent for publication of this case report. It is available upon request.
